# Morphometric Changes of the Corpus Callosum in Congenital Blindness

**DOI:** 10.1371/journal.pone.0107871

**Published:** 2014-09-25

**Authors:** Francesco Tomaiuolo, Serena Campana, D. Louis Collins, Vladimir S. Fonov, Emiliano Ricciardi, Giuseppe Sartori, Pietro Pietrini, Ron Kupers, Maurice Ptito

**Affiliations:** 1 Auxilium Vitae Volterra, Volterra, Italy; 2 Department of General Psychology, University of Padua, Padua, Italy; 3 McConnell Brain Imaging Centre, Montreal Neurological Institute, McGill University, Montreal, QC, Canada; 4 Laboratory of Clinical Biochemistry and Molecular Biology, Department of Surgery, Medical, Molecular, and Critical Area Pathology, University of Pisa, Pisa, Italy; 5 MRI Lab, Fondazione Toscana ‘G. Monasterio’, Pisa, Italy; 6 Clinical Psychology Branch, Pisa University Hospital, Pisa, Italy; 7 Harland Sanders Chair in Visual Science, École d’optométrie, Université de Montréal, Montréal, Québec, Canada; 8 BRAINlab, Department of Neuroscience and Pharmacology, Panum Institute, University of Copenhagen, Copenhagen, Denmark; University of Regensburg, Germany

## Abstract

We examined the effects of visual deprivation at birth on the development of the corpus callosum in a large group of congenitally blind individuals. We acquired high-resolution T1-weighted MRI scans in 28 congenitally blind and 28 normal sighted subjects matched for age and gender. There was no overall group effect of visual deprivation on the total surface area of the corpus callosum. However, subdividing the corpus callosum into five subdivisions revealed significant regional changes in its three most posterior parts. Compared to the sighted controls, congenitally blind individuals showed a 12% reduction in the splenium, and a 20% increase in the isthmus and the posterior part of the body. A shape analysis further revealed that the bending angle of the corpus callosum was more convex in congenitally blind compared to the sighted control subjects. The observed morphometric changes in the corpus callosum are in line with the well-described cross-modal functional and structural neuroplastic changes in congenital blindness.

## Introduction

Over the past few decades, studies in animals and humans have given ample evidence that the absence of vision from birth causes profound structural changes in the brain. These changes take place not only in the visually deprived cortex but also in other brain areas [1], [2]. Recent studies on congenital blindness have shown that the deprived visual cortex undergoes widespread structural and metabolic changes, and becomes activated by a large variety of non-visual stimuli, suggesting the presence of cross-modal plastic rearrangements [1], [3]. For example, compared to normal sighted subjects, the occipital cortex of congenitally blind subjects shows strong volumetric reductions in striate and extra-striate visual areas and also in non-visual areas such as the hippocampus [4], [5], an increase in cortical thickness in striate cortex [3], [6], [7], increased resting-state metabolism in the occipital cortex [1], [8], and changes in functional and anatomical connectivity [9]–[15]. In addition, the retino-thalamo-cortical pathway is strongly reduced in congenitally blindness [5], [16], [17]. Beyond the visual cortex, structural changes have also been reported in the hippocampus [4], [18], the corticospinal tract [19] and the posterior part of the corpus callosum [5], [17], [20], [21].

There is strong evidence from animal studies that visual deprivation affects the development of the posterior callosal connections. For example, dark rearing from birth exacerbates the partial elimination of callosal projections, leaving only a few axons at the border of area 17/18 [22], [23]. Similarly, bilateral eyelid suture in kittens at birth leads to a 50% reduction in the number of callosal neurons [24]. Monocular enucleation at birth, on the other hand, produces an abnormally wide distribution of callosal cells at the 17/18 border, that is reminiscent of the effects obtained with convergent or divergent strabismus, or monocular eyelid suture [25]. Hence, all these manipulations of visual input at birth produce a widespread distribution and an exuberant number of callosal terminals in animals [25], [26]. In sharp contrast with the converging animal data, the purported neuroplastic changes in the corpus callosum following visual deprivation in humans remain a matter of debate. For instance, two studies [5], [21] reported that the posterior part of the corpus callosum is reduced, whereas the area from the genu to the anterior body of the corpus callosum is increased in congenitally blind humans [5]. The latter region contains small-to-medium sized fibers that mainly connect prefrontal cortical areas [27]. However, two other studies [2], [28] failed to find volumetric changes in the anterior and posterior part of the corpus callosum area in a small and heterogeneous group of early blind and anophthalmic individuals. The discrepancies between these studies may be due to differences in the type of patients, onset of blindness (late versus early versus since birth), sample size and the methodology used to compute brain volumetry.

In this study, we investigated the corpus callosum in a large and homogeneous cohort of congenitally blind subjects, using a method that enables us to take into account the between group global shape differences in the corpus callosum. We showed previously that shape is an important factor for correctly subdividing and measuring the volume of the sub-callosal regions [29].

## Materials and Methods

### Subjects

Twenty-eight congenitally blind individuals, (16 males, age: 37±16 yrs, range: 19–60 yrs; 12 females, age: 33±13 yrs, range: 19–63 yrs) and 28 normal sighted control subjects matched for gender and age (16 males, age: 31±8 yrs, range: 22–48 yrs; 12 females: 33±13 yrs, range: 21–58 yrs) were included in the study. Participants were recruited from three different centers (see below). All subjects gave written informed consent after the protocol was read to them and the study protocol was approved by the Ethical Committee of Copenhagen and Frederiksberg municipalities, the Comité mixte d’éthique de la recherché/Regroupement Neuroimagerie Québec (CMER-RNQ) and the Comitato Etico per la Sperimentazione del Farmaco dell’Università di Pisa. All blind participants were deprived of vision from birth and had no history of light perception. None of the participants had any known neurological, psychiatric or genetic syndromes. Demographic data and the causes of blindness are summarized in Table 1.

**Table 1:** Demographic data and causes of blindness.

Subject ID	Sex	Age	Handedness	Characteristics blindness	
				Cause	Onset
cb01	F	49	R	Retinopathy of prematurity	Birth
cb02	M	41	R	Retinis pigmentosa	Birth
cb03	M	39	R	Retinal detachment	Birth
cb04	M	58	R	Congenital cataract	Birth
cb05	M	38	R	Retinopathy of prematurity	Birth
cb06	F	31	R	Glaucoma, aniridia	Birth
cb07	M	20	R	Leber’s amaurosis	Birth
cb08	M	23	R	Congenital cataract	Birth
cb09	M	27	R	Fibroblasia	Birth
cb10	F	27	R	Optic nerve atropy	Birth
cb11	F	42	R	Retinopathy of prematurity	Birth
cb12	M	60	R	Congenital glaucoma	Birth
cb13	F	31	R	Microphthalmia + Congenital Cataract	Birth
cb14	F	23	R	Optic nerve atropy	Birth
cb15	M	35	R	Retinopathy of prematurity	Birth
cb16	M	57	R	Congenital cataract	Birth
cb17	M	58	R	Congenital glaucoma	Birth
cb18	F	19	R	Congenital glaucoma	Birth
cb19	F	63	R	Congenital glaucoma	Birth
cb20	F	26	R	Retinopathy of prematurity	Birth
cb21	M	56	R	Retinopathy of prematurity	Birth
cb22	F	21	R	Retinopathy of prematurity	Birth
cb23	M	21	R	Retinopathy of prematurity	Birth
cb24	F	41	R	Retinopathy of prematurity	Birth
cb25	M	19	R	Retinopathy of prematurity	Birth
cb26	M	23	L	Retinopathy of prematurity	Birth
cb27	F	27	R	Retinopathy of prematurity	Birth
cb28	M	23	R	Glaucoma	Birth

Abbreviations: M = male; F = female; R = right; L = left.

### Imaging

T1-weighted MRIs of the brain were collected at the Unité de Neuroimagerie Fonctionelle, Université de Montréal, Canada (8 blind and 8 sighted controls), the MRI Lab, Fondazione Toscana ‘G. Monasterio’, Italy (11 blind and 11 sighted controls) and the DRCMR, Hvidovre University Hospital, Copenhagen, Denmark (9 blind and 9 sighted controls). The Montreal MRIs were acquired on a 1.5 Tesla magnet (Magnetom Avanto, Siemens, Erlangen, Germany), equipped with an 8-channel head coil, using a gradient echo pulse sequence (TR = 2240 ms, TE = 9.2 ms, FOV = 256 mm, matrix = 256×256, voxel size = 1 mm^3^). The Pisa MRIs were acquired on a GE Signa 1.5 Tesla scanner (General Electric Milwaukee, WI), equipped with a 2-channel head coil, using high resolution T1-weighted spoiled gradient echo (TR = 2270 ms, TE = 3.6 ms, FOV = 240 mm, matrix = 512×512, voxel size = 0.5×0.5×1 mm; resampled to 1 mm^3^). The Copenhagen MRIs were acquired on a Siemens Trio 3 Tesla magnet (Siemens, Erlangen, Germany), equipped with an 8-channel head coil, using a gradient echo pulse sequence (TR = 1540, TE = 3.9 ms; flip angle = 30°; FOV = 256 mm; matrix = 256×256; voxel size = 1 mm^3^).

### Pre-processing of the T1-MRI images

Native brain MRI volumes were checked for scanner artefacts and gross anatomical abnormalities such as enlarged ventricles and/or macroscopic focal lesions. Subsequently, images were corrected for image intensity non-uniformity [30] and intensity normalized to a range from 0 to 100. Each pre-processed native MRI brain volume was individually re-aligned by positioning it along the anterior-posterior commissure line and by rotating it such that the septum pellucidum and at least a large part of the falx were visible in the sagittal plane. The high spatial resolution brain sampling allowed images to be rotated by small angles in the three orthogonal planes, thus facilitating the identification of anatomical landmarks for the selection of the regions of interest (ROI). This is particularly important in order to obtain a reliable measure of the corpus callosum that was sampled from its most medial part on a 1 mm thickness slice.

### Segmentation of the corpus callosum

We manually segmented the corpus callosum using the mid-sagittal MRI slice in which the septum pellucidum and the falx were simultaneously visible. The pericallosal sulcus was used as the dorsal and rostral boundaries, whereas the III ventricle and the cisterna superior formed the ventral boundary. In cases where it was not possible to distinguish between the corpus callosum and the fornix, the boundary was defined by the shortest line between the anterior and the posterior end of the corpus callosum with the fornix. Two independent raters (SC and FT), who were unaware of the participants’ condition, drew the ROIs. In case of disagreement, a consensus was sought. ROIs were mapped using the interactive program Display, developed at the McDonald Brain Imaging Centre, Montreal Neurological Institute (http://www.bic.mni.mcgill.ca/ServicesSoftware/ServicesSoftwareMincToolKit). This program permits labelling of voxel regions on single slices of the MRI volume and allows for the simultaneous visualisation in the sagittal, axial and coronal planes. Any area can be selected using the Display “mouse brush”, to colour the voxels of the ROI. This colouring procedure, accompanied with the 3D view of the MRI planes, allows better landmark identification. We first determined the ROI anatomical landmarks, and then we marked the voxels of the ROI by colouring.

### Shape analysis

To quantify the shape of the corpus callosum, we applied the same procedure as used in our previous investigations [29], [31]. An automatic procedure constructed the minimum rectangle covering each individual’s identified ROI on native space MRI. The bending angle of the corpus callosum was measured by calculating the value of the vertex angle of the isosceles triangle which had the same base and height of the minimum rectangle circumscribing the corpus callosum using the equation: [2arctg/(base/2height)].

### Brain volume normalization

#### i. Preprocessing

After non-uniformity correction and intensity normalization described above, all MRIs scans were linearly transformed into the Talairach-like space MNI152 2009c [32] using the mritotal program described by Collins et al. [33]. Brain tissues were segmented with BEaST [34] and intensities within brain only were normalized linearly using histogram matching.

#### ii. Population-specific template

**Figure 1:**
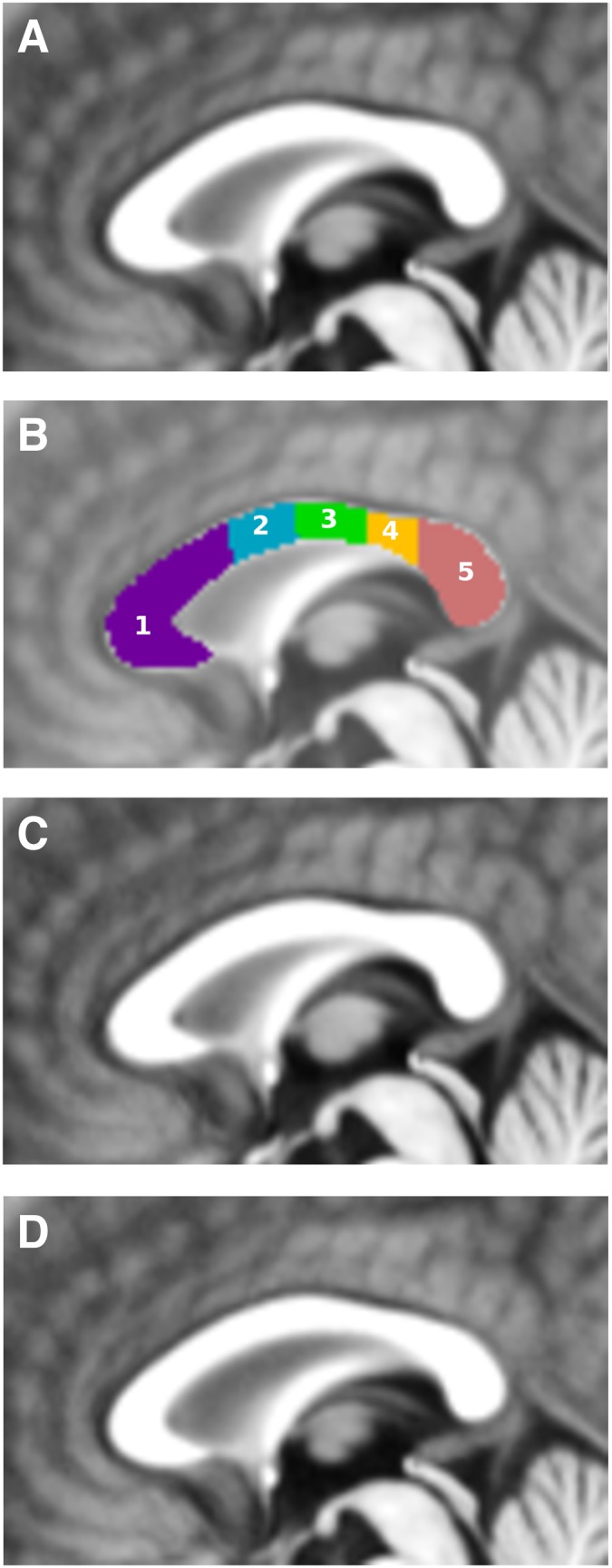
Mid-sagittal sections of the corpus callosum. A: callosal area of the anatomical average of both cohorts; B: callosal sub-regions drawn by two experts (FT and LC). This resulted in the following regions: 1) the anterior third of the corpus callosum, including the rostrum, the genu, and the rostral body; 2) the anterior mid-body; 3) the posterior mid-body; 4) the isthmus; and 5) the splenium; C: callosal area of the anatomical average of NC cohort; D: callosal area of the anatomical average of CB cohort. Note the smaller splenium in CB.

An unbiased, left-right symmetric, group average was then created using the algorithm described in Fonov et al. [32]. This procedure builds the population-specific average anatomical template – (see Figure 1A) in an iterative fashion. An initial group average template is built with voxel-by-voxel averaging all linearly transformed datasets. At each iteration 1) the non-linear mapping between each dataset and the group average is estimated with ANIMAL [35] the transformation is used to resample the dataset into the average space, 3) all datasets are then averaged together to create a temporary template, and 4) the inverse of the average deformation of all datasets is applied to the temporary template to create a spatially unbiased average template. The first iterations estimate large deformations to account for large morphological differences between subjects. Each successive iteration reduces the spacing between grid nodes that define the transformation to estimate finer transformations to refine the fit between the subject and the evolving average template. When completed, the procedure yields an unbiased average template of all datasets and the non-linear transformations required to map each subject from their native space to the common average template space.

Two of the authors (FT, LC) drew the CC labeling on the sagittal slice of the unbiased average template that best represented the mid-sagittal section according to criteria previously described for individual CC labeling, see Figure 1B, 1C. For the purpose of visualization, averages of two cohorts (blind and sighted) were also calculated – see Figure 1D for average of NC and 1E for CB cohort.

### Sub-regional areal segmentation of the average template

We used an automatic procedure to construct the minimum rectangle that circumscribes the corpus callosum. Four lines perpendicular to the longest side of the minimum rectangle subdivided the corpus callosum into five contiguous sub-regions, covering 33, 17, 17, 13 and 20% respectively of its total length. Since the automatic procedure could not properly subdivide the rostrum, genu and rostral body of the corpus callosum as proposed by Witelson [36], we kept the regions in the anterior third callosal together as in Bermudez & Zatorre [37]. This division resulted in the following contiguous sub-regions along the rostro-caudal direction: a) the anterior third of the corpus callosum, including the rostrum, the genu, and the rostral body; b) the anterior mid-body; c) the posterior mid-body; d) the isthmus; and e) the splenium.

### Automatic measurement of cross-section area

Transformation fields calculated during the group average creation process were used to calculate the surface area of each labeled sub-region by integrating Jacobian determinant maps of the transformation fields in the sagittal plane, producing a measurement cross sectional area of each CC region in linearly transformed stereotaxic space, thus normalizing for brain size. Multiplying the Jacobian determinant by the inverse of the linear native-to-stereotaxic space transform scaling factor allows us to estimate the native (absolute) area of the CC region in subject.

### Data Analysis

To test for possible differences between MRI data collection sites (Copenhagen, Montreal and Pisa), we used a one-way ANOVA on the callosal ROIs in native space. No significant differences were found (F(2,53) = 0.05, n.s.). We used an ANCOVA to assess group and gender differences in the bending angle of the corpus callosum in native MRI space, thereby including age as a co-varying factor. Finally, we used ANCOVAs to test for group and gender differences in the overall surface area of the corpus callosum and in the surface areas of the five callosal sub-regions, including age as a co-varying factor. These ANCOVA analyses used the surface areas estimated in the common stereotaxic space to account for differences in brain size. Bonferroni correction was used for multiple comparisons of the five callosal subregions, with p-values≤0.01 considered as statistically significant. Post-hoc comparisons were carried out by Scheffe’s test; significance level was set at p<0.05. Data are presented as means ± SD.

## Results

### Shape analysis

**Figure 2:**
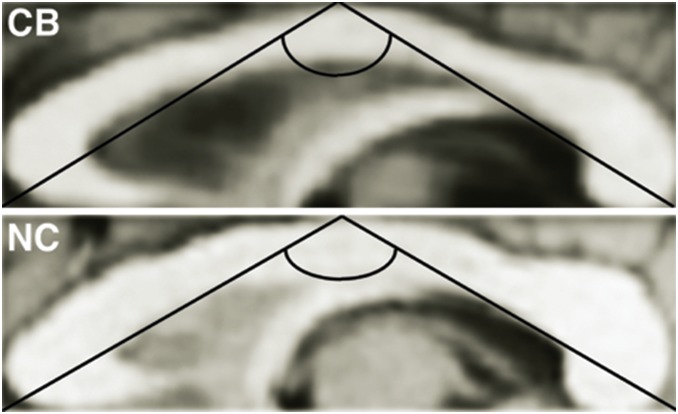
Example of a shape analysis of the corpus callosum in a congenitally blind (CB) and a normal sighted control (NC) individual. An automatic procedure generated the minimum rectangle that circumscribes the corpus callosum. The vertex angle of the isosceles triangle was estimated, which has the same base and height of the minimum rectangle circumscribing the corpus callosum. Congenitally blind subjects had a more convex corpus callosum compared to normal control subjects. Note the group difference in ratio between height and base.

As illustrated in Figure 2, the corpus callosum binding angle was more convex in congenitally blind than in sighted controls (F_(1,52)_) = 6.77; p = 0.012, CB: 109.6°±4.9° vs. NC: 113.4°±5.7°). We did not find a significant gender difference or group by gender interaction effect.

### Sub-regional areas

**Figure 3:**
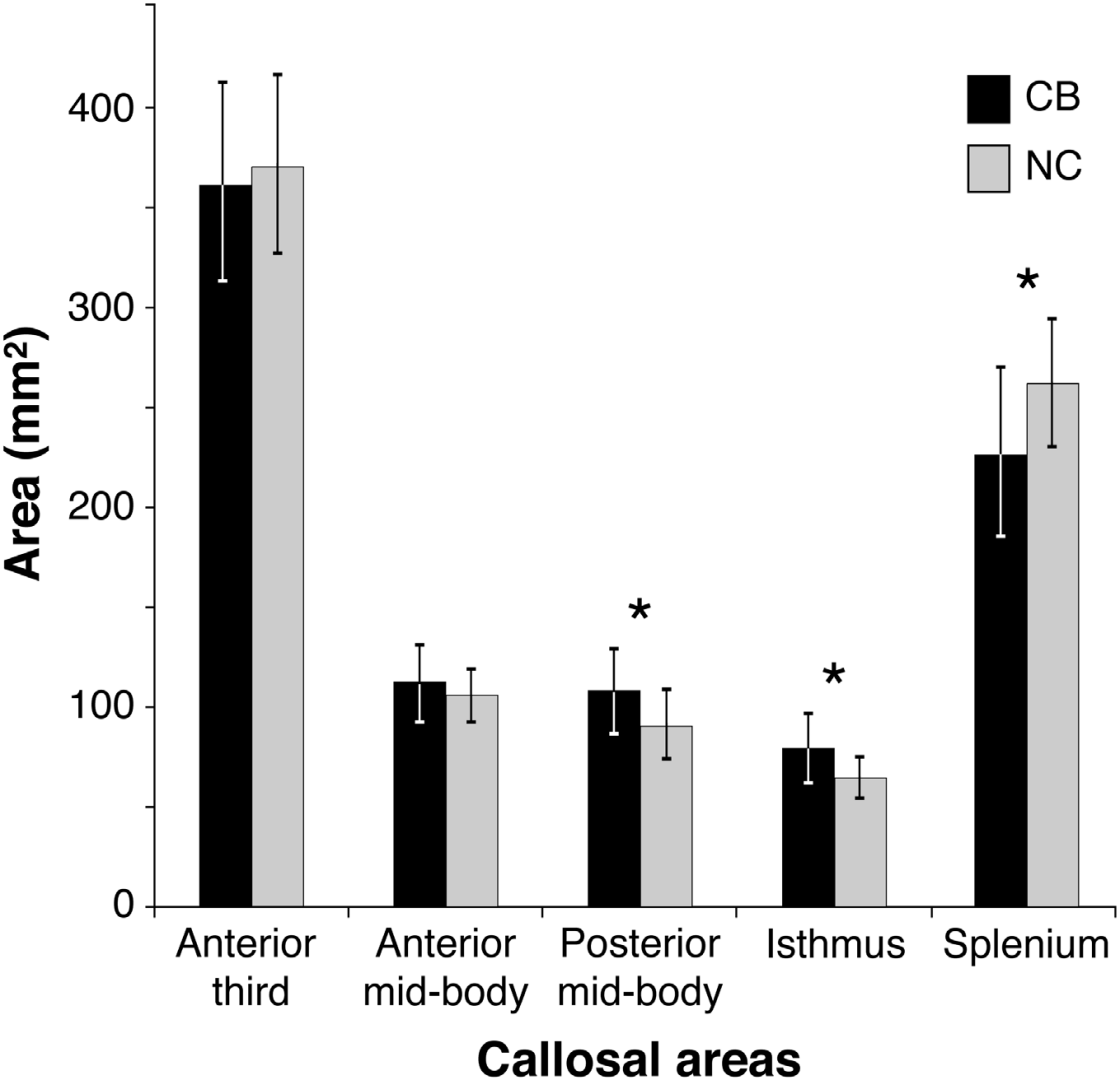
Callosal sub-region areas in congenitally blind (CB) and normal sighted control (NC) subjects. CB had a significant decrease in surface area of the splenium but a significant increase in the caudal part of the body and the isthmus.

The total corpus callosum surface area for the two groups was not significantly different. However, we observed significant group differences for three of the five callosal sub-regions, all belonging to the posterior half of the corpus callosum (Figure 3). In line with our hypothesis, blind individuals had a significantly lower surface area of the splenium (F(1,54) = 12.64, p<0.001; NC: 260.84±41.7 mm^2^ vs CB: 225.64±31.7 mm^2^). In addition, blind individuals also had a significant increase in the size of the posterior mid-body (107.26±21.67 mm^2^ and 89.64±16.51 mm^2^ for blind and sighted individuals, respectively; F(1,54) = 11.7, p = 0.001) and the isthmus (79.4±16.66 mm^2^ and 64.16±10.31 mm^2^ for blind and sighted individuals, respectively; F(1,54) = 14.61, p<0.001). Finally, we observed a significant group by gender interaction for the isthmus (F_(1,52)_ = 7.24, p = 0.01). A post-hoc analysis revealed that blind males had a larger isthmus than any of the other groups (CB males: 87.24±18.81 mm^2^; CB females: 68.95±11.83 mm^2^; NC males: 63.49±10.05 mm^2^; NC females: 65.06±11.04 mm^2^). None of the other contrasts were significant.

## Discussion

The callosum is a crucial pathway, transferring information between the two hemispheres. A body of data from neuroanatomical, electrophysiological, tracing and partial ablation studies in nonhuman primates has revealed that the corpus callosum is topographically organized [38]. This was confirmed in several recent neuroimaging studies in humans, using diffusion tensor imaging and fiber tractography [39]–[46]. These studies have shown that also in humans specific functions are conveyed through different sub-divisions of the corpus callosum. Thus, fibers crossing the rostrum and the genu of the corpus callosum convey taste information, whereas those in the rostral body, anterior mid-body and posterior mid-body are involved in motor functions and touch. Finally, callsoal fibers of the isthmus and splenium carry auditory and visual information.

In this study, we examined the corpus callosum in a large and homogeneous cohort of congenitally blind individuals who never had any visual experience, and who were devoid of focal brain damage, hydrocephalus or specific genetic syndromes. We used a specific method to split the corpus callosum into sub-callosal areas, ruling out possible errors induced by group differences in shape [29]. Our results showed significant group differences in callosal shape and volume.

### Shape differences in the corpus callosum

Our data indicate that the corpus callosum has a more convex bending angle in congenitally blind compared to sighted subjects. We ascertained that this difference in callosal shape could not be attributed to associated neurological or psychiatric diseases [29], [47]–[49]. A very convex callosal shape was reported in patients affected by severe brain atrophy [29], [50] while a very concave shape was observed in patients with premature termination of brain development [31], [47]. As a significant number of our congenital blind subjects suffered from retinopathy of prematurity, it could be argued that the observed alteration in callosal shape is due to preterm birth. To rule out this possibility, we subdivided our CB sample into those with blindness due to retinopathy of prematurity (N = 12) and those with blindness due to other aetiologies (N = 16). This statistical comparison did not yield a significant difference, indicating that prematurity per se was not responsible for the change in callosal binding.

How to interpret changes in callosal shape? There is evidence that a multitude of neurological diseases will cause atrophy of the corpus callosum, leading to changes in its size and shape. Loss or reductions of cortical neuronal populations will lead to a reduced number of inter-hemispheric connections, whereas lesions of the white matter will induce alterations in the recipient cortical layers. This will cause alterations in both the size and the shape of the corpus callosum. This idea is supported by numerous studies that have shown that various brain pathologies such as schizophrenia [51], Niemann-Pick Disease [48], multiple sclerosis [52] and “diffuse axonal injuries” [53] result in modifications of the inter-hemispheric commissure. In the case of congenital blindness, the atrophy of the visual cortex [5] will cause a reduction in the number of fibers coursing through the splenium, inducing both a volume reduction of that sub-region and a generalized change in callosal shape. Alterations in callosal shape are even used as a clinical index of the severity of brain lesions [48].

Group differences in callosal shape could theoretically induce measurement errors when sub-dividing the callosum in different parts [31], [54]. A more convex shape (similar to that in CB) could lead to an underestimation of the surface area of the median part of the corpus callosum, i.e. the anterior mid-body, the posterior mid-body and the isthmus; at the same time, it could lead to an overestimation of the anterior and posterior part of the corpus callosum [29]. To rule out this possibility, we used a non-linear transformation of each brain volume to ensure that our results would not be affected by possible group differences in shape [29], [55].

### Surface area differences in the corpus callosum

In order to estimate callosal surface areas, we used integration of the jacobian determinants of the transformation fields obtained during the group average creation process described above. This method reduces the inter-individual variability in gross brain size and allows for optimal inter-subject comparison of ROI sizes. Our specific intra-callosal area measurement approach revealed that the group differences were constrained to the posterior half of the corpus callosum. In line with our hypothesis, the splenium, i.e. the area connecting large areas of the occipito-temporal and occipito-parietal cortices, was significantly smaller in the group of blind subjects. This finding is in line with earlier voxel-based-morphometry reports of volume reductions of striate and extrastriate visual cortical areas in congenitally blind individuals [5], [16], [56] and with reports of decreased functional connectivity of the left and right occipital cortices in congenital blind individuals, as measured by resting state fMRI [12]. Together, this has prompted us to speculate that visual deprivation triggers a cascade of structural changes, going from grey matter reductions in the lateral geniculate nucleus, the striate and extrastriate visual cortices, up to white matter reductions in the posterior part of the corpus callosum that relays interhemispheric transfer of visual or acoustic information. Despite this significant reduction in the volume of the splenium in congenitally blind individuals, around the vast majority of the splenial connections remain present [28]. This raises the issue as to the type of information that these fibers convey. Numerous studies have shown that congenital blindness in humans leads to a reorganization of the brain through a process of cross-modal plasticity [57]. Indeed, the visual cortex of congenitally blind individuals can be activated by a wide variety of non-visual inputs and using various cognitive tasks [57]–[59]. In addition, several studies have shown that there are also alterations in the functional connectivity of the occipital cortex [9]–[13], [60]. Together, these findings may explain the observation that there is only a partial volumetric loss in the splenial volume.

In contrast with the reduction in splenial surface, congenitally blind individuals had a 20% increase in the volume of the caudal mid-body and isthmus. Whereas the posterior mid-body is involved in the interhemisheric transfer of somatosensory information from primary and secondary somatosensory cortex, and posterior parietal cortex, the isthmus contains fibers crossing the midline originating from posterior parietal and superior temporal areas [40]. These data are in line with a vast literature showing that blind individuals rely more strongly on auditory and somatosensory information, and perform better in various tactile and auditory tasks [1].

## Conclusion

Our results show that congenital blindness leads to a selective reduction in the surface area of the splenium, and to an increase in surface area of the isthmus and the posterior part of the body. Further studies based on histological evaluation and diffusion tensor imaging will provide more specific information about the type and the origin of the fibres running in different parts of the corpus callosum in congenitally blind individuals. Such studies will further our knowledge of how inter-hemispheric connectivity in congenital blindness differs from that in normal control subjects, thereby indicating the relationship between brain developmental plasticity and cognitive functions.
